# Four Days Are Enough to Provide a Reliable Daily Step Count in Mild to Moderate Parkinson’s Disease through a Commercial Smartwatch

**DOI:** 10.3390/s23218971

**Published:** 2023-11-04

**Authors:** Edoardo Bianchini, Silvia Galli, Marika Alborghetti, Lanfranco De Carolis, Alessandro Zampogna, Clint Hansen, Nicolas Vuillerme, Antonio Suppa, Francesco E. Pontieri

**Affiliations:** 1 Department of Neuroscience, Mental Health and Sensory Organs (NESMOS), Sapienza University of Rome, 00189 Rome, Italy; edoardo.bianchini@uniroma1.it (E.B.); silvia.galli@uniroma1.it (S.G.); marika.alborghetti@gmail.com (M.A.);; 2 AGEIS, Université Grenoble Alpes, 38000 Grenoble, France; nicolas.vuillerme@univ-grenoble-alpes.fr; 3 Department of Human Neurosciences, Sapienza University of Rome, 00185 Rome, Italy; alessandro.zampogna@uniroma1.it (A.Z.); antonio.suppa@uniroma1.it (A.S.); 4 Department of Neurology, Kiel University, 24105 Kiel, Germany; c.hansen@neurologie.uni-kiel.de; 5 LabCom Telecom4Health, Orange Labs & Université Grenoble Alpes, CNRS, Inria, Grenoble INP-UGA, 38000 Grenoble, France; 6 Institut Universitaire de France, 75005 Paris, France; 7 IRCCS Neuromed Institute, 86077 Pozzilli, Italy

**Keywords:** gait, IMU, Parkinson’s disease, sensors, smartwatch, step count, reliability, wearable, activity monitor, digital health technology, m-health

## Abstract

Daily steps could be a valuable indicator of real-world ambulation in Parkinson’s disease (PD). Nonetheless, no study to date has investigated the minimum number of days required to reliably estimate the average daily steps through commercial smartwatches in people with PD. Fifty-six patients were monitored through a commercial smartwatch for 5 consecutive days. The total daily steps for each day was recorded and the average daily steps was calculated as well as the working and weekend days average steps. The intraclass correlation coefficient (ICC) (3,k), standard error of measurement (SEM), Bland–Altman statistics, and minimum detectable change (MDC) were used to evaluate the reliability of the step count for every combination of 2–5 days. The threshold for acceptability was set at an ICC ≥ 0.8 with a lower bound of CI 95% ≥ 0.75 and a SAM < 10%. ANOVA and Mann–Whitney tests were used to compare steps across the days and between the working and weekend days, respectively. Four days were needed to achieve an acceptable reliability (ICC range: 0.84–0.90; SAM range: 7.8–9.4%). In addition, daily steps did not significantly differ across the days and between the working and weekend days. These findings could support the use of step count as a walking activity index and could be relevant to developing monitoring, preventive, and rehabilitation strategies for people with PD.

## 1. Introduction

Parkinson’s disease (PD) is a progressive neurodegenerative disease characterized by motor and non-motor symptoms [1]. Bradykinesia (slowness of movement), rigidity (increased muscle stiffness), and rest tremor are the three cardinal motor manifestations in PD, but gait and balance disturbances are rather common, particularly with disease progression [2].

Gait is an essential motor activity and its disturbances have a profound impact on functional independence, disability, quality of life, and mortality in older adults [3,4,5]. Gait impairments in PD include continuous alterations such as reduced step length and speed, increased cadence and variability, diminished arm swing, and episodic disturbances such as freezing of gait (FOG) [6]. Alterations in biomechanics and the disorders associated with the disease make walking an energetically unfavorable activity in PD [7], and can severely affect a patient’s quality of life and functional autonomy [8], increasing the risk of falls [9].

Daily steps are rather easy to collect and represent a useful measure of ambulatory activity and mobility [10]. More importantly, daily step count has been reported to be associated with general mortality [5,11,12,13,14] as well as with the incidence of several pathological conditions such as cancer [15], cardiovascular disease [15], and dementia [16] in older adults. In PD patients, previous studies have reported an association between the degree of disease severity [17] and average physical activity [18].

The spread of commercial devices, such as smartphones and smartwatches [19], has made step counting even more accessible, and high interest is growing in sensor-based mobility assessment in healthy adults [20] and several diseases [21], including PD patients [22]. However, a critical point is whether these commercial devices are sufficiently accurate, reliable, and valid for measuring steps. Step counting algorithms are usually designed to work with people with physiological walking patterns, therefore PD could represent a challenge. Motor symptoms increase the background noise of the recording leading to an inaccurate estimate of the number of steps particularly if the device is worn on the wrist [23,24,25]. We have recently demonstrated the accuracy of a commercial smartwatch in measuring steps against manual step counting when the smartwatch was worn on the side least affected by the disease [23]. Furthermore, a recent work from Ginis and colleagues [26] demonstrated criterion validity for average daily steps measured using commercial smartwatches against a research-grade inertial sensor.

When measuring step count in real-world conditions, the number of days required for monitoring daily steps is another crucial aspect to ensure a reliable estimate of the variable of interest that could be representative of habitual walking behavior. Furthermore, it must be considered whether daily steps are collected on consecutive days or random days since the latter has been reported to reduce the reliability of step counting [27]. Indeed, in previous work from Kang et al. [27], 5 consecutive days were sufficient to reliably estimate the daily step count in healthy older adults through a research-grade, wrist-worn, inertial sensor, while 6 days were needed when using non-consecutive days. This aligns with previous studies reporting that a period from 2 to 7 consecutive days can accurately predict walking activity in healthy older adults [28,29,30,31,32]. Other works also reported that a monitoring window of 2 consecutive days, irrespective of the type of day, could be sufficient in patients with disability from neurologic diseases, such as multiple sclerosis [33] and poststroke patients [34]. In PD, a limited number of studies have investigated the minimum number of days required to achieve a reliable estimate of the average daily steps in a real-world setting [35], using an ankle-mounted, research-grade inertial sensor (step activity monitor [36]) in 92 mild to moderate PD patients, for 7 consecutive days. The authors concluded that 2 days of monitoring were sufficient to obtain good reliability in estimating daily steps, as indicated by an intraclass correlation coefficient (ICC) (2,1) > 0.9. Notwithstanding, ankle-mounted devices are generally less tolerated by users [37,38] compared with wrist-worn sensors [37,39,40] and research-grade devices are usually expensive, less available on the market, and require a higher level of expertise to be used [19], thus limiting their application. To this end, the use of commercial, wrist-worn devices, such as smartwatches, could help disseminate this technology and increase the possibility of monitoring walking at home. However, no study has investigated the minimum number of monitoring days required to achieve a reliable estimate of the average daily steps measured in an unsupervised, real-world setting, through a commercial smartwatch in PD patients.

## 2. Materials and Methods

This was a cross-sectional study aimed at investigating the minimum number of days needed to reliably estimate daily step count using a commercial smartwatch, in an unsupervised, real-world setting, in people with mild to moderate PD.

The study was performed in accordance with the ethical standards as laid down in the 1964 Declaration of Helsinki and its later amendments. Approval was granted by the local Ethics Committee. Data collection and processing followed the current European regulation for data protection. All participants provided written informed consent before the start of measurements.

Participants were longitudinally recruited in the Movement Disorder Outpatient Service of the Sant’Andrea University Hospital, Rome, Italy, in the period between March 2023 and July 2023. The inclusion criteria were as follows: (i) diagnosis of idiopathic PD according to MDS criteria [41]; (ii) age 18 years or older; (iii) disease stage < 4 according to the modified Hoeh and Yahr scale (mHY) (“severe disability; still able to walk or stand unassisted”) [42]; (iv) ability to walk independently without walking aids; (v) ability to perform the experimental procedure. The exclusion criteria were as follows: (i) cognitive impairment as defined by a Montreal Cognitive Assessment (MoCA) score < 24; (ii) orthopedic, rheumatologic, or systemic conditions affecting mobility as judged by the assessor.

Participants were evaluated during scheduled visits on Wednesday, Thursday, or Friday, at the hospital. Demographics (age, sex) and anthropometric measures (weight, height, body mass index (BMI)) were collected. Disease duration, disease stage according to the modified Hohen and Yahr scale (mHY), and Levodopa Equivalent Daily Dose (LEDD) [43], were also collected. The Movement Disorder Society—Unified Parkinson’s Disease Rating Scale (MDS-UPDRS)—part III [26] was used to assess motor symptoms severity. The MDS-UPDRS—part IV was used to assess the presence of motor complications.

Participants received the smartwatch Garmin Vivosmart 4 (Garmin, Olathe, KS, USA), to be worn at home for at least 5 days, including at least one weekend day, on the wrist on the side least affected by the disease, following a previous study from our group [23]. The smartwatch was configured according to the producer’s recommendations [44] by indicating the users’ age, height, weight, and the wrist on which the smartwatch was worn (i.e., left or right). Patients were asked to perform daily activities as usual. From the smartwatch dashboard, the total daily steps for each day was recorded. The average daily steps was calculated as well as the working days (i.e., Monday to Friday) and weekend days (i.e., Saturday and Sunday) average steps.

The statistical analyses were performed using JASP v0.17.2.1 (JASP Team, University of Amsterdam), R v4.3.1, and RStudio v2023.06.0+421 for Windows (R Foundation for Statistical Computing, Vienna, Austria). Descriptive statistics were calculated for the examined variables. The normality of the distributions was assessed by histogram and residual plots inspection. A one-way analysis of variance (ANOVA) was performed to assess the difference in the daily step count, across the 5 days. To evaluate the relative reliability for each combination of 2–5 days, a two-way intraclass correlation coefficient (ICC) with a fixed set of raters and averaged ratings was used (ICC (3,k), where k was the number of days of measurement), together with a custom R script. The following reference cut-off values for ICC interpretation were used [45]: Excellent: >0.90; Good: 0.75–0.90; Moderate: 0.50–0.75; Poor: <0.50. The a priori threshold for an acceptable ICC was set at a point estimate ≥ 0.80 with a lower bound of 95% confidence interval (CI 95%) ≥ 0.75. Standard error of measurement (SEM) and minimal detectable change with a confidence interval of 95% (MDC95) were used to compute the absolute reliability for all combinations of 2–4 days [46] and were reported as absolute value and percentage of criterion measure (SEM% and MDC95%, respectively). The criterion was the average daily step count derived from the 5 days. SEM% values lower than 10% were considered acceptable [47]. Bland–Altmann plots were used to assess the average magnitude bias between the 2–4 days combinations and criterion as well as 95% limits of agreement, calculated as bias ± 1.96 SD. For all analyses, the significance threshold was set at α < 0.05. All data were reported as mean ± SD or median (Q1–Q3) for numerical data and N (%) for categorical variables.

## 3. Results

A total of 56 PD patients were enrolled in the study. All patients were monitored through Garmin Vivosmart 4 at home for a period of 5 consecutive days. Participants took on average 5861 ± 3086 daily steps ranging from 357 to 12,509. Details of demographic, anthropometric, and clinical variables are shown in Table 1.

Details of the daily steps, working and weekend day steps, and average daily steps across the 5-day period are shown in Table 2.

**Figure 1:**
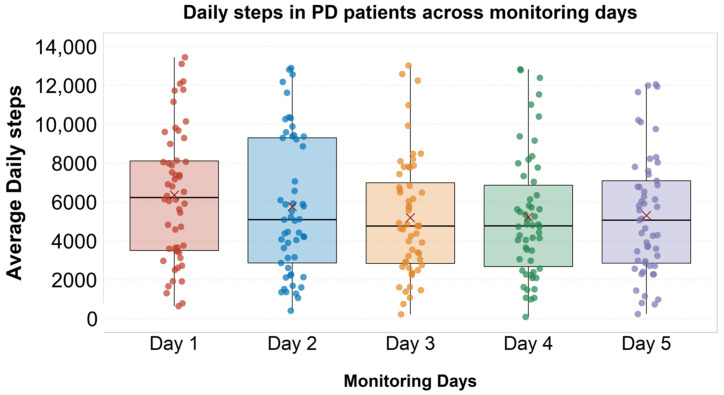
Boxplots showing daily steps for each monitoring day in PD patients. Thick line in the boxes indicates the median; lower and upper box limits indicate first (Q1) and third quartile (Q3), respectively; black vertical lines indicate lower and upper outliers boundaries calculated as Q1 − (1.5 × IQR) and Q3 + (1.5 × IQR), respectively. Red X indicates mean values for each disease stage. PD: Parkinson’s disease.

The ANOVA test showed no significant differences in the daily steps across the 5 days (F_2,272_ = 1.997; *p* = 0.095) (Figure 1).

**Figure 2:**
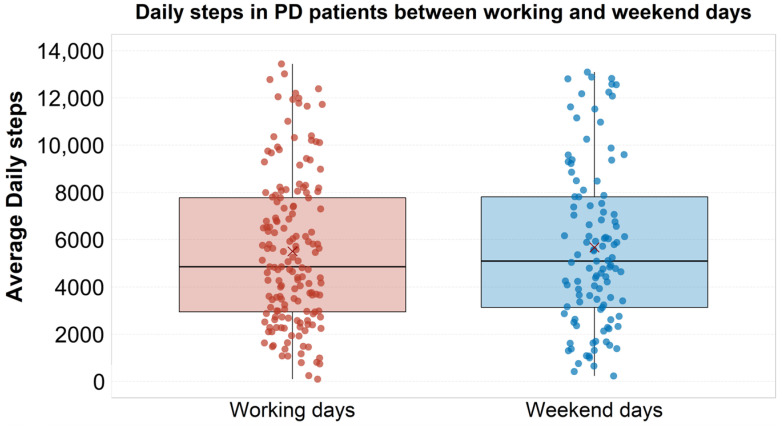
Boxplots showing daily steps for working and weekend days in PD patients. The thick line in the boxes indicates the median; lower and upper box limits indicate first (Q1) and third quartile (Q3), respectively; black vertical lines indicate lower and upper outliers boundaries calculated as Q1 − (1.5 × IQR) and Q3 + (1.5 × IQR), respectively. Red X indicates mean values for each disease stage. PD: Parkinson’s disease.

The Mann–Whitney test showed no significant difference between the working days and weekend days for daily steps (W = 9051.52; *p* = 0.774) (Figure 2).

Table 3 shows the ICC (3,k) values for all the combinations of 2–5 days and Bland–Altman statistics, SEM, and MDC for all the combinations of 2–4 days.

**Figure 3:**
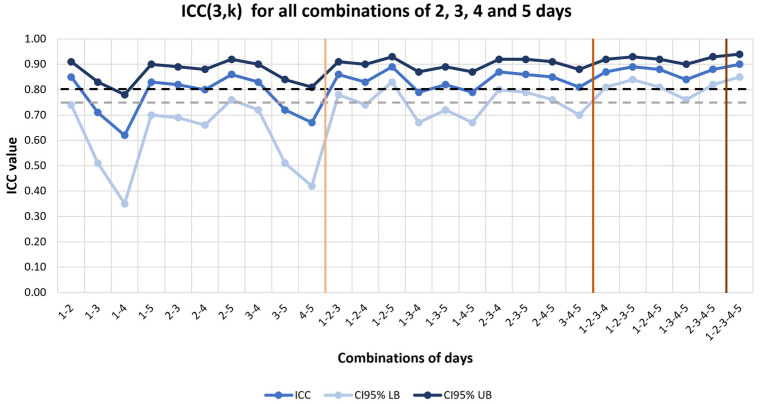
Graph showing the ICC(3,k) values across the different combinations of monitoring days. The top dark blue line represents 95% CI upper bound. The middle blue line represents the ICC (3,k) point estimate. The bottom light blue line represents 95% CI lower bound. Vertical brown lines were added to mark 2-day, 3-day, 4, day, and 5-day combinations. Dashed horizontal dark and light grey lines were added to mark the 0.8 threshold for the point estimate and the 0.75 threshold for 95% CI lower bound, respectively. CI: confidence interval; ICC: intraclass correlation coefficient. LB: lower bound; UB: upper bound.

The consecutive 5-day recording showed an excellent ICC. All the 4-day combinations, both consecutive and non-consecutive, demonstrated a good ICC above the predefined criterion, a SEM below 10% and a small bias from the Bland–Altmann statistics. Two- and three-day combinations did not consistently show an ICC above the a priori threshold (Figure 3).

## 4. Discussion

This cross-sectional study aimed at investigating the minimum number of days needed to reliably estimate the daily step count using a commercial smartwatch, in an unsupervised, real-world setting, in people with mild to moderate PD. To our knowledge, this is the first study to investigate this aspect using a commercial smartwatch in PD patients.

We conclude the following: (i) 4 days are needed to reliably estimate the average daily steps in mild to moderate PD, and (ii) daily steps did not differ across the 5 monitoring days and between working days and weekend days. These results are discussed in detail in the following sections.

### 4.1. Reliability of Smartwatch-Based Average Daily Steps in PD Patients

We found that a minimum of 4 days was needed to reliably estimate the average daily steps in mild to moderate PD using a consumer-grade, wrist-worn smartwatch.

Only one study has examined this aspect before in a similar group of 92 people with PD (Age: 67.3 ± 7.8 years; females: 41/92 (45%); mHY: 2.3 ± 0.6) [35]. In this report, the authors concluded that two days of monitoring were sufficient to obtain a reliable daily step count estimate with an ICC (2,1) >0.9, using a research-grade, ankle-mounted step counter (step activity monitor, SAM) worn for 7 continuous days [35].

The lower minimum required number of days found in this study could be due to the different devices used and the position where the instrument was worn. It is generally expected that an ankle-mounted device achieves higher reliability than a wrist-worn one [36]. Such research-grade devices undergo rigorous validation processes, and have displayed high accuracy and reliability under a broad range of walking conditions in different diseases, including PD [36,48,49]. Previous studies on patients with different neurological conditions, such as multiple sclerosis [33] and post-stroke [34], also reported that 2 days could be sufficient to reliably estimate daily steps using an arm-mounted research-grade device (SenseWear Armband [50]). However, in line with our results, other studies reported a minimum number of monitoring days of 3–7 in healthy elderly patients using hip- and wrist-mounted devices [28,29,30,31]. In PD, motor symptoms could hamper the performance of the step detection algorithm. In particular, the step detection performances of wearable sensors have been reported to be lower with a reduced gait speed [51,52,53] and increased cadence [54], both features of a parkinsonian gait, and during discontinuous walking periods [25] that likely occur in PD patients due to reduced automaticity and episodes of FOG. Moreover, bradykinesia and reduced arm swing during ambulation could lead to an underestimation of the number of steps [25], whereas tremor and dyskinesia may induce overestimation of the step count [23,24], particularly when the device is worn on the wrist [24]. Furthermore, inter- and intra-day symptom fluctuations [55] and increased gait variability are common aspects of PD [6]. Indeed, motor complications were present in 27% of our patients. All these elements and the use of a research-grade device could explain why the minimum number of days required to reliably estimate the average daily steps was higher in our study than in the previous literature on other neurological conditions.

The result that 4 days of monitoring was sufficient to obtain a reliable average daily step count is extremely relevant, as shorter monitoring periods could make it easier to collect step counts in daily clinical practice. Additionally, this is the first study to report Bland–Altman statistics, SEM, and MDC95 for daily step count in PD patients. The Bland–Altman plots revealed a good agreement in terms of magnitudes of bias and 95% limits of agreement for the 4-day combinations; the SEM showed that the 4-day combinations reached an absolute reliability within the a priori threshold of 10% (Table 3). The 4-day combinations also showed the lowest degree of MDC95, with the values ranging from 1198 to 1524 steps/day (20–26% of the criterion). MDC is defined as the minimal change that falls outside the measurement error of an instrument used to collect a given parameter [46,56]. This metric is extremely relevant to design future studies aiming to assess the effectiveness or cost/effectiveness of interventions, since it allows the calculation of the sample size. In our study, with 4-day monitoring windows, a difference of 1366 steps, on average, could not be attributed to random variations of the measurements, in mild–moderate PD patients measured through a commercial smartwatch.

All these findings could allow a wider implementation of step count as a reliable and easy-to-collect index of ambulatory activity and help in designing future studies including daily step count as an outcome measure in PD patients. As walking is commonly impaired in PD, it is indispensable to be able to quantify habitual walking in this population on a daily basis to develop preventive, educational, and rehabilitation strategies.

### 4.2. Difference in Daily Steps across Days and between Working and Weekend Days

In our study, participants took on average 5861 ± 3086 daily steps. This result is in line with previous reports showing average daily step values between 4500 and 7000 in similar PD populations [26,57,58,59]. There was no significant difference across the five days and between the working and weekend days. The non-significant effect of the day on average daily steps is supported by previous research on healthy elderly individuals [31] and patients with other diseases showing a moderate level of disability, such as rheumatoid arthritis [60] and multiple sclerosis [33,61]. Regarding the difference between the working and weekend days, our results are in line with previous work from Benka Wallén and collaborators on mild to moderate PD patients [57] and other reports in older adults [31,62,63]. The majority of PD patients are diagnosed after the age of 60 [2], close to or after the retirement age in Italy, and it has been reported that PD patients retire 4–7 years earlier than the general population [64]. In our PD population, 44 out of 56 (nearly 80%) of the patients were retired; therefore, a difference between the working and weekend days in terms of walking pattern may not have been evident. However, our finding are in line with previous studies which also reported no physical activity and step count difference in healthy working adults between working and weekend days [65,66]. Conversely, Paul and colleagues [35], showed that PD patients take more steps on working days than on the weekend. However, it must be noted that, in that study, the difference in steps between the working and weekend days was rather small (around 600 steps/day; around 7% of the total reported daily steps), and the authors reported that they were only able to identify the time participants spent walking with moderate or greater intensity; therefore, this aspect could have influenced their results [35]. Taken together, our findings support the idea that patients with PD and moderate disability display relatively consistent levels of ambulatory activity across the week, as identified by the average daily steps.

### 4.3. Limitations

We acknowledge that this study has limitations. First, the PD patients included in our study had, on average, relatively good levels of physical ability and cognitive functions, due to the inclusion and exclusion criteria. This could, hence, limit the generalization of our results and future studies enrolling PD participants with lower functional scores, greater disease severity, and more severe cognitive impairment are encouraged and included in our plans. Second, we monitored the patients for 5 days only, therefore we could not draw firm conclusions regarding longer monitoring windows. However, previous studies in neurological patients used a 5-day time window to monitor daily steps and applied similar data analysis methods [33], and our results allowed us to confirm the reliability of a 4-day monitoring period for step counting. Third, the patients received a smartwatch on working days (i.e., Wednesday, Thursday, or Friday) and this could have affected our results, particularly regarding the difference between working and weekend days. However, we could hypothesize a minimal impact of this aspect on our results since our results are in line with the previous literature and we did not find any difference across the monitoring days. Fourth, we found that with a 4-day monitoring period, a difference of 1366 steps, on average, could not be attributed to random variations of the measurements. Although this aspect is relevant to design future studies, no report, to date, has investigated the minimal clinically important difference for daily steps in PD patients; therefore, no conclusions regarding the clinical relevance of a change in daily steps could be drawn and future studies will need to clarify this aspect. Finally, we tested only one smartwatch in our study, therefore more studies are needed to generalize our results and validate commercial smartwatches in clinical populations.

## 5. Conclusions

In mild to moderate PD patients, a minimum of 4 monitoring days was needed to achieve a reliable estimate of the average daily steps through a commercial smartwatch (Garmin Vivosmart 4). The step count did not significantly differ across the monitoring days and between the working and weekend days. Taken together, the short monitoring period required and the finding that PD patients with a moderate disability displayed relatively consistent levels of ambulatory activity across the week could facilitate a broader implementation of step count as an index of walking activity in this population. The present findings could be highly relevant to develop monitoring, preventive, educational, and rehabilitation strategies for people with PD.

## Figures and Tables

**Table 1:** Demographic, anthropometric, and clinical characteristics of the enrolled population. BMI: body mass index; F: females; LEDD: Levodopa equivalent daily dose; M: males; MDS-UPDRS-III: Movement Disorder Society Unified Parkinson’s Disease Rating Scale part III; mHY: modified Hoehn and Yahr scale; PD: Parkinson’s disease.

PD Patients (N = 56)		
Age (years)		69.5 ± 7.8
Retired		44 (79%)
Height (cm)		174 ± 7.6
Weight (kg)		79.0 ± 13.0
BMI (kg/m^2^)		26.2 ± 3.8
Sex	M	43 (77%)
	F	13 (23%)
Disease duration (years)		7.1 ± 4.7
LEDD (mg)		604 ± 325
mHY		2 (2–2.5)
Motor complications		15 (27%)
MDS-UPDRS-III		29 (23–32)

**Table 2:** Daily steps for each monitoring day, average steps during working and weekend days, and average daily steps across the 5-day period in the enrolled population. ANOVA: Analysis of variance; PD: Parkinson’s disease.

PD Patients (N = 56)		
Steps day 1	6858 ± 4230	
Steps day 2	6280 ± 4094	
Steps day 3	5203 ± 3034	F_2,272_ = 1.997; *p* = 0.095 ^a^
Steps day 4	5401 ± 3411	
Steps day 5	5520 ± 3436	
Working days	5805 ± 3693	W = 9051.52; *p* = 0.774 ^b^
Weekend days	5932 ± 3719	
Average daily steps	5861 ± 3086	

^a^ One-way ANOVA, ^b^ Mann–Whitney U test.

**Table 3:** ICC (3,k) for all combinations of 2–5 days and Bland–Altman statistics, SEM, and MDC for all combinations of 2–4 days. ICC values are reported as point estimate (95%CI lower bound—95% CI upper bound). Bland–Altmann statistics are reported as bias (upper limit of agreement; lower limit of agreement). SEM and MDC are reported as absolute number of steps (percentage of criterion measure). ICC and SEM values within the a priori thresholds for acceptability are displayed in bold. CI: confidence interval; ICC: intraclass correlation coefficient; MDC95: minimum detectable change with 95% CI; MDC95%: minimum detectable change with 95% CI expressed as percentage of criterion measure SEM: standard error of measurement; SEM%: standard error of measurement expressed as percentage of criterion measure.

Combinations of Days	ICC (3,k)	Bland–Altmann Statistics	SEM (SEM%)	MDC95 (MDC95%)
Days 1-2	0.85 (0.74–0.91)	−707.9 (−3340.2; 1924.4)	1510 (25.8)	4185 (71.4)
Days 1-3	0.71 (0.51–0.83)	−169.3 (−2337.2; 1998.6)	1109 (18.9)	3074 (52.5)
Days 1-4	0.62 (0.35–0.78)	−269.3 (−2195.3; 1656.7)	1010 (17.2)	2801 (47.8)
Days 1-5	0.83 (0.70–0.90)	−361.1 (−3245.7; 2523.6)	1503 (25.6)	4165 (71.1)
Days 2-3	0.82 (0.69–0.89)	119.8 (−1829.7; 2069.3)	993 (16.9)	2752 (47.0)
Days 2-4	0.80 (0.66–0.88)	15.6 (−2312.2; 2343.4)	1177 (20.1)	3263 (55.7)
Days 2-5	**0.86 (0.76–0.92)**	−76.3 (−2353.2; 2200.7)	1154 (19.7)	3198 (54.6)
Days 3-4	0.83 (0.72–0.90)	578.3 (−2492.3; 3648.8)	1657 (28.3)	4592 (78.4)
Days 3-5	0.72 (0.51–0.84)	508.4 (−1488.0; 2504.8)	1130 (19.3)	3133 (53.5)
Days 4-5	0.67 (0.42–0.81)	368.3 (−1844.4; 2581.0)	1337 (22.8)	3705 (63.2)
Days 1-2-3	0.86 (0.78–0.91)	−252.5 (−1678.8; 1173.9)	764 (13.0)	2118 (36.1)
Days 1-2-4	0.83 (0.74–0.90)	−320.5 (−1609.8; 968.7)	726 (12.4)	2014 (34.4)
Days 1-2-5	**0.89 (0.83–0.93)**	−381.7 (−2422.9; 1659.4)	1100 (18.8)	3050 (52.0)
Days 1-3-4	0.79 (0.67–0.87)	46.6 (−1466.6; 1559.8)	767 (13.1)	2125 (36.3)
Days 1-3-5	0.82 (0.72–0.89)	−7.3 (−1565.0; 1553.4)	789 (13.5)	2187 (37.3)
Days 1-4-5	0.79 (0.67–0.87)	−93.0 (−1413.5; 1227.4)	674 (11.5)	1869 (31.9)
Days 2-3-4	**0.87 (0.80–0.92)**	237.9 (−1685.2; 2161.0)	1001 (17.1)	2775 (47.3)
Days 2-3-5	**0.86 (0.79–0.92)**	184.0 (−1112.1; 1480.0)	681 (11.6)	1887 (32.2)
Days 2-4-5	**0.85 (0.76–0.91)**	94.1 (−1394.5; 1582.6)	758 (12.9)	2102 (35.9)
Days 3-4-5	0.81 (0.70–0.88)	492.6 (−1288.0; 2273.1)	1026 (17.5)	2845 (48.5)
Days 1-2-3-4	**0.87 (0.81–0.92)**	−72.1 (−968.0; 823.7)	**459 (7.8)**	1271 (21.7)
Days 1-2-3-5	**0.89 (0.84–0.93)**	−114.4 (−1177.9; 949.1)	**550 (9.4)**	1524 (26.0)
Days 1-2-4-5	**0.88 (0.81–0.92)**	−175.3 (−1068.2; 717.6)	**484 (8.3)**	1342 (22.9)
Days 1-3-4-5	**0.84 (0.76–0.90)**	109.7 (−717.1; 936.5)	**432 (7.4)**	1198 (20.4)
Days 2-3-4-5	**0.88 (0.82–0.93)**	252.1 (−879.4; 1383.6)	**539 (9.2)**	1495 (25.5)
Days 1-2-3-4-5	**0.90 (0.85–0.94)**	-	-	-

## Data Availability

The data presented in this study are available on reasonable request from the corresponding author.
